# Halothane: Is there still any place for using the gas as an anesthetic?

**Published:** 2011-07-01

**Authors:** Ali Dabbagh, Samira Rajaei

**Affiliations:** 1 Anesthesiology Research Center, Shahid Beheshti University of Medical Sciences, Tehran, IR Iran; 2 Immunology Department, School of Medicine, Tehran University of Medical Sciences, Tehran, IR Iran

**Keywords:** Halothane, Nanotechnology, Anesthesiology, Hepatitis

## Abstract

The fluorinated hydrocarbons that are used for anesthesia are derived from ether. Although they have many benefits, there are several side effects of these drugs, including untoward hepatic effects. Whether the use of halothane gas can be revitalized is unknown. Introducing nanocarriers inside the halothane molecule can increase its benefits as an anesthetic in the lungs and cardiovascular system and prevent exposure to the liver. The findings of new fields, such as cancer therapy, and anesthetic agents, such as propofol, can improve the quality of the drug using nanomedicine.

## 1. Introduction

The fluorinated hydrocarbons that are used for anesthesia are derived from ether. Despite their benefit, there are several side effects of these drugs. One of these agents halothane, a true innovation in the pharmacology of anesthesia, was introduced in the 1950s by Charles Suckling, a British chemist, over 2 years of research and retesting[1][2][3]. James Raventon evaluated the pharmacological characteristics of the drug, and finally, Michael Johnston, a famous physician and anesthesiologist, described its clinical superiorities. Not too many years were needed to recognize the first clinical presentation of a side effect, later termed halothane-induced hepatitis, in a 39-year-old woman in 1958[3][4][5]. This occurrence became a major trigger for the production of less harmful anesthetic gases[5].

There are 2 types of this reaction to halothane involving the liver: type 1, or mild hepatitis, is accompanied by increased levels of plasma transaminases and is a self-limited process[6][7]; type 2 is severe hepatotoxicity[6][7][8], associated with acute hepatic failure and mortality. The most likely mechanism for this type of disease is immune related, occurring after the generation of free radicals, which are the end products of halothane hepatic metabolism[8]. There is a lower risk of liver toxicity after consumption of newer halogenated anesthetics, such as enflurane, isoflurane, sevoflurane, and desflurane[8], since they have lower metabolism rates in the liver. But, compared to halothane, these agents are much more expensive[6][7][8]. In other words, halothane has been supplanted by its younger "brothers and sisters" at a higher cost. The liver related complications of these agents, although they have decreased, have not disappeared, while the effects of halothane on the pulmonary and cardiovascular system have vanished. Can halothane gas be reintroduced as an anesthetic and bronchodilative agent that does not have untoward effects on the liver?

## 2. The hypothesis

During the last few years, nanoparticles have been increasingly a matter of interest in nearly all aspects of medicine, and much evidence has emerged regarding nanotechnology in medicine[9][10]. This field is an evolving one that has seen many improvements, which are the result of its implications in medicine. One of the important issues that is considered in this field is the use of nanocarriers or nanomachines, which constitute a nanoscale delivery system. These delivery systems help prevent the unwanted exposure of tissues to a drug[11][12]. The issue is that halothane is an anesthetic agent with a relatively long history of clinical use as an anesthetic with beneficial cardiopulmonary effects and as a potent bronchodilator[3][4][5][6]. Also, the drug is widely recognized and used in many parts of the world[6][7][8]. If the halothane molecule could be altered in such a way that it could pass the lung and reach the brain and other organs, except for the liver, the new molecule could have all the useful effects of an inhalational anesthetic agent without causing major hepatic side effects. This could be the basis for producing halothane with such properties into which nanocarriers are inserted[13][14]. Since halothane is a well-known inhaled anesthetic that is used in many operating rooms, this nano-halothane would have a tremendous impact on the clinical practice of anesthesia [15].

## 3. Discussion of the hypothesis

There are many opportunities in which we can use current and emerging nanotechnologies to produce entirely novel classes of therapeutics[16]. There has been some progress in many fields, such as cancer therapy[17], and in producing new anesthetic agents, such as propofol[12], which are all accompanied by special challenges. Of course, the problem with drugs like halothane is its elimination from hepatic uptake; usually, nanoproducts are absorbed in the liver[18]. Yet, it seems that there are many methods that can be used to circumvent this problem, such as pegylation of drug molecules. Producing a new molecule of halothane can create prevent its untoward effects and while maintaining its benefits as an anesthetic agent.
